# Assessing the Real-World Safety of Regadenoson for Myocardial Perfusion Imaging: Insights from a Comprehensive Analysis of FAERS Data

**DOI:** 10.3390/jcm14061860

**Published:** 2025-03-10

**Authors:** Xingli Xu, Qian Guo, Yaxing Li, Chungang Zhai, Yang Mao, Yanling Zhang, Lei Zhang, Yun Zhang

**Affiliations:** State Key Laboratory for Innovation and Transformation of Luobing Theory, Key Laboratory of Cardiovascular Remodeling and Function Research of MOE, NHC, CAMS and Shandong Province, Department of Cardiology, Qilu Hospital of Shandong University, Jinan 250012, China; xuxingli623@163.com (X.X.); guoqianmail@163.com (Q.G.); 19588908672@163.com (Y.L.); zhaichungang87@sdu.edu.cn (C.Z.); maoyangs@163.com (Y.M.); 15692315052@163.com (Y.Z.)

**Keywords:** regadenoson, FAERS, disproportionality analysis, adverse event, real-world safety

## Abstract

**Background/Objectives**: Regadenoson, a selective adenosine A_2A_ receptor agonist, is primarily prescribed for myocardial perfusion imaging (MPI). As its clinical use becomes more widespread in practice, assessing its safety in real-world settings is essential. **Methods**: In this research, disproportionality analysis was applied to evaluate the safety of Regadenoson by examining all adverse event (AE) reports since 2004 in the FDA Adverse Event Reporting System (FAERS), in which Regadenoson was identified as the primary suspected drug. The reporting odds ratio (ROR), proportional reporting ratio (PRR), multi-item gamma Poisson shrinker (MGPS), and Bayesian confidence propagation neural network (BCPNN) were used to analyze AEs associated with Regadenoson. The Weibull distribution was utilized to model the temporal risk of AEs. **Results**: The results confirmed some known adverse reactions, such as nausea, shortness of breath (dyspnea), palpitations/vomiting, headache, dizziness, chest pain, and flushing (facial redness or warmth), which were also listed on the drug’s label. New potential adverse reactions not mentioned in the label were identified, including micturition urgency, mental status changes, conversion disorder, eye movement disorder, and genital paraesthesia. This study highlighted the significance of monitoring AEs, particularly right after the start of Regadenoson administration. **Conclusions**: This study provides preliminary safety data on Regadenoson’s real-world use, corroborating known adverse effects while uncovering new potential risks. These findings offer valuable safety insights for clinicians when prescribing Regadenoson for the use of MPI.

## 1. Introduction

Regadenoson, a selective adenosine A_2A_ receptor agonist, represents a major advancement in cardiovascular medicine [1]. By inducing coronary vasodilation, it effectively mimics the effects of exercise on increasing myocardial blood flow (MBF) to the heart, making it an invaluable tool in myocardial perfusion imaging (MPI) [2]. The growing prevalence of coronary artery disease (CAD) has imposed a substantial economic burden on healthcare systems and society at large [3]. The clinical use of Regadenoson allows for the accurate assessment of CAD and myocardial perfusion, providing critical insights into heart function and aiding in the diagnosis and management of cardiovascular conditions [4].

Regadenoson is the first U.S. Food and Drug Administration (FDA)-approved selective A_2A_ adenosine receptor agonist currently in clinical use. It stands out as a preferred coronary vasodilator for MPI due to its rapid onset and short duration of action, convenience of use as a fixed-dose bolus rather than based on body weight, and excellent safety profile [1]. Numerous clinical trials have demonstrated its safety even in patients with reactive airway disease [5,6,7,8]. Although its side effects can be rapidly reversed with an antagonist, if necessary, they still warrant careful consideration [1].

The FDA Adverse Event Reporting System (FAERS) is a database established by the U.S. FDA to collect and analyze the reports of adverse events (AEs) related to drugs [9]. It helps the U.S. FDA to identify potential drug safety risks. Recently, FAERS was used as a crucial drug safety monitoring tool to identify AEs associated with various drugs and medical products, including the long-term use of selective serotonin reuptake inhibitors in cardiovascular risk, psychiatric disorders related to immune checkpoint inhibitors, as well as dupilumab in the potential risk of eosinophilic pneumonia [10,11,12].

This study evaluated the safety of Regadenoson in real-world clinical settings by analyzing data from the FAERS database using disproportionality analysis. The results are intended to offer clinicians valuable insights regarding the safety and risk factors associated with the use of Regadenoson in practice. By identifying potential AEs and providing a clearer understanding of its safety, this study seeks to guide professionals in making informed decisions when administering Regadenoson to patients.

## 2. Materials and Methods

### 2.1. Data Sources

This study utilized the original data from the FAERS database, a publicly accessible non-mandatory reporting system where reports are mainly contributed by consumers, health professionals, pharmacists, physicians, and others. The analysis encompassed all AE reports in the format of the original ASCII data package, where Regadenoson was identified as the main suspected drug, with the period covering from the first quarter of 2004 to the third quarter of 2024.

### 2.2. Data Management and Study Design

**Figure 1:**
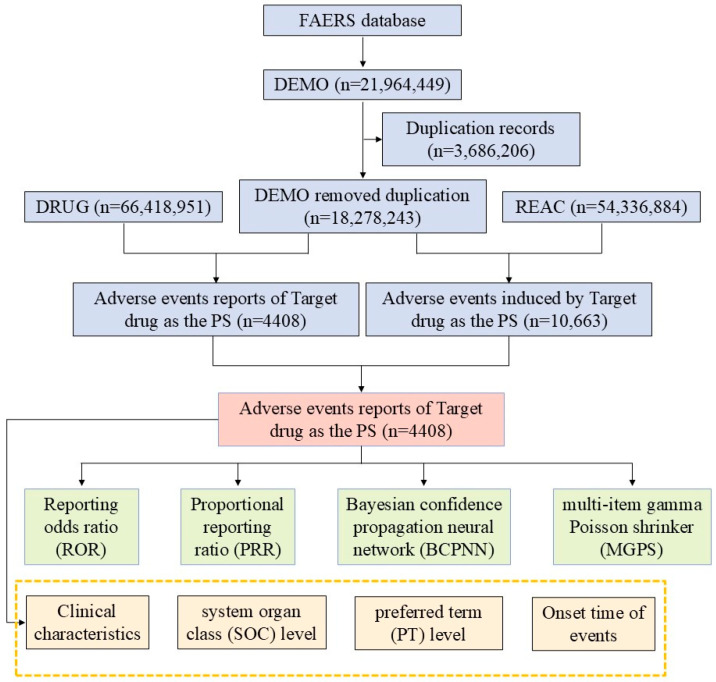
Flowchart demonstrating the AE analysis process for Regadenoson using the FAERS database. AE, adverse event; DEMO, demographic data; DRUG, drug; FAERS, FDA Adverse Event Reporting System; REAC, reaction data; ROR, reporting odds ratio; PRR, proportional reporting ratio; BCPNN, Bayesian confidence propagation neural network; MGPS, multi-item gamma Poisson shrinker; SOC, system organ class; PS, primary suspect; PT, preferred term.

In managing the data, data and report deduplication and the standardization of the AE terminology were performed. Firstly, duplicate reports were removed based on the FDA-recommended method. Specifically, the reports were sorted by case identifiers (CASEIDs), FDA receipt date (FDA_DT), and the unique identifier for each report (PRIMARYID). For reports with the same CASEIDs, the one with the latest FDA_DT was retained. If both CASEID and FDA_DT were identical, the report with the largest PRIMARYID was kept. Secondly, starting from the first quarter of 2019, deduplicated reports were removed based on the CASEID values in the deletion report list, as each quarterly data package includes such a list of deleted reports. Thirdly, the latest version of the *Medical Dictionary for Regulatory Activities* (MedDRA) dictionary (MedDRA 27.1) is applied to code the AE terms in the FAERS database, including the preferred terms (PTs) and the corresponding system organ class (SOC) classifications. These latest versions are then used for subsequent analysis. A comprehensive flowchart outlining the study design is presented in Figure 1.

### 2.3. Statistical Analysis

Descriptive analysis was conducted to summarize the characteristics of AE reports related to Regadenoson. To identify potential signals of adverse reactions, four disproportionality analysis methods were utilized, including the reporting odds ratio (ROR) [13], proportional reporting ratio (PRR) [14], Bayesian confidence propagation neural network (BCPNN) [15], and multi-item gamma Poisson shrinker (MGPS) [16]. The threshold of the Medicines and Healthcare products Regulatory Agency (MHRA) combined standard method is applied as the threshold for the PRR method in signal detection.

An AE was considered as a potential adverse reaction if it exceeded the positivity threshold in at least one of these methods. Detailed two-by-two contingency tables are shown in Supplementary Table S1. The formulas and thresholds for the disproportionality analysis are provided in Supplementary Table S2. The onset time of Regadenoson-related AEs was defined by the time interval between the reported occurrence of AEs (from the DEMO file) and the start of Regadenoson treatment (from the THER file). To model the temporal changes in AE incidence, the Weibull distribution was applied. All analyses were conducted using the SAS software version 9.4.

## 3. Results

### 3.1. Clinical Characteristics

This research included 4408 reports of AEs (totaling 10,663 AEs) where Regadenoson was identified as the suspected primary agent (Figure 1). Among these, 47.75% of the cases involved female patients, and 32.44% involved male patients. The age group of ≥65 years accounted for the largest proportion (35.69%), followed by the age group of 45–64 years (18.04%). A majority of the reports were submitted by consumers (51.7%), with 23.32% from healthcare professionals and 18.9% from pharmacist. The reports of severe and non-severe cases each account for 56.35% and 43.65%. The occurrence of AEs was primarily concentrated between 2012 and 2018, followed by a year-on-year decline in cases after 2018. However, in 2024, there was a sudden increase, with the number rising to 350 cases. Most reports originated from the United States (98%). More details are provided in Table 1.

### 3.2. Distribution of AEs at the SOC Level

**Figure 2:**
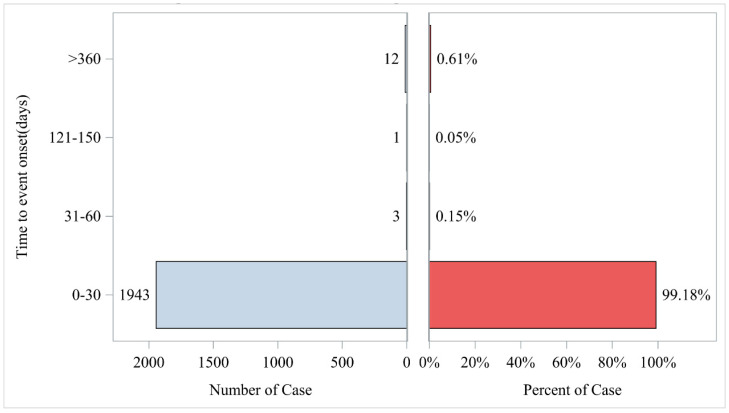
Time to event report distribution of AE reports. AE, adverse event.

AEs related to Regadenoson contained 26 of the 27 significant SOCs. As shown in Table 2, notable findings were identified in various categories, including but not limit to nervous system disorders; general disorders and administration site conditions; gastrointestinal disorders; cardiac disorders; investigations; respiratory; thoracic and mediastinal disorders; vascular disorders; musculoskeletal and connective tissue disorders; injury, poisoning, and procedural complications; as well as skin and subcutaneous tissue disorders. The signal strength of Regadenoson-related AEs at the SOC level in the FAERS database is illustrated in Table 2 and Figure 2.

### 3.3. Distribution of AEs at the PT Level

AEs related to Regadenoson were analyzed based on frequency and evaluated for potential safety signals. Among the top 50 most common AEs, the known common side effects include nausea, shortness of breath (dyspnea), palpitations/vomiting, headache, dizziness, chest pain, and flushing (facial redness or warmth). The serious adverse effects (rare but possible) include severe hypotension, cardiac arrest, seizures, bradycardia (slow heart rate), and loss of consciousness. Furthermore, additional AEs not currently listed on the product label were identified, including micturition urgency, mental status changes, conversion disorder, eye movement disorder, and genital paraesthesia. Detailed information regarding the top 50 most frequent AEs for Regadenoson at the PT level is shown in Table 3. Representative classic and non-classic AEs of Regadenoson and their clinical implications are shown in Table 4. All AEs meeting the criteria for a positive signal of Regadenoson at the PT level are listed in Supplementary Table S3.

### 3.4. Onset Time of AEs

**Figure 3:**
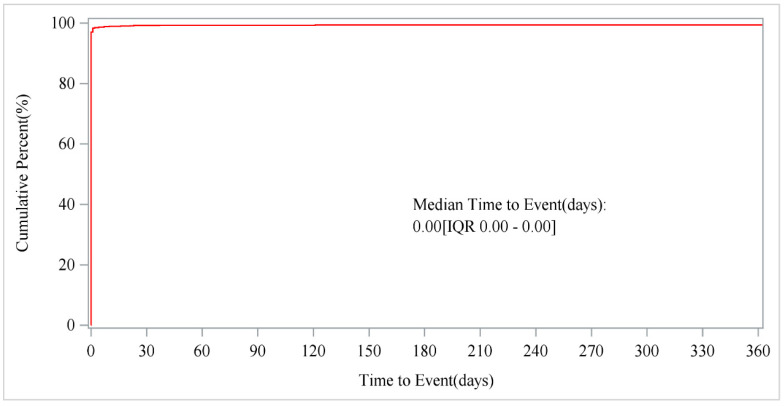
Cumulative incidence of AEs. AE, adverse event.

After excluding unreliable reports, a total of 1959 AEs associated with Regadenoson provided the onset time data. Almost all AEs occurred right after the start of Regadenoson administration (*n* = 1943, 99.18%). The median onset time was 0 days (interquartile range [IQR] 0.00–0.00 days). However, rare cases occurred at 2 months (*n* = 3, 0.15%), 6 months (*n* = 1, 0.05%), and 12 months (*n* = 12, 0.61%). The timeline of these events is shown in Figure 2. The cumulative incidence curve of AEs is presented in Figure 3.

## 4. Discussion

This investigation conducted a thorough assessment of AEs related to Regadenoson after its 2008 market introduction. Through analyzing the FAERS database, this study confirmed the common occurrence of AEs listed on the drug’s label, such as nausea, dyspnea, vomiting, headache, dizziness, chest pain, flushing, as well as the serious adverse effects, including severe hypotension, cardiac arrest, seizures, bradycardia, and loss of consciousness. Moreover, AEs not found on the label, such as micturition urgency, mental status changes, conversion disorder, eye movement disorder, and genital paraesthesia, were also revealed. These findings highlight the importance of vigilant monitoring of patients, particularly immediately following Regadenoson administration and over an extended period, as delayed AEs, although rare, have been reported. Such monitoring is essential for the timely management and mitigation of potential side effects. Further investigation is warranted to better understand the mechanisms and risk factors associated with these delayed AEs.

Currently, single-photon emission computed tomography (SPECT) and positron emission tomography (PET) MPI are widely utilized, accounting for approximately 50% of diagnostic procedures in the evaluation of patients with suspected CAD [17]. These imaging techniques require the use of vasodilators, such as adenosine, dipyridamole, adenosine triphosphate, and Regadenoson. Adenosine, a commonly used non-selective agonist of A_1_, A_2A_, A_2B_, and A_3_ receptors, is associated with a range of AEs including negative chronotropic, dromotropic, and inotropic effects, as well as anti-β-adrenergic effects, mast cell degranulation, and bronchoconstriction [18]. In addition, Regadenoson is the only FDA-approved selective A_2A_ adenosine receptor agonist, offering several advantages such as rapid onset, short duration of action, and fixed-dose administration [1]. It is generally well tolerated, with a lower incidence of side effects such as chest pain, shortness of breath, and flushing, making it a safer alternative [1]. However, some AEs are still associated with its clinical use. It is crucial for clinicians to be aware of these potential risks and to take appropriate measures for the management of Regadenoson.

The landmark ADVANCE-MPI study revealed that the primary cardiovascular side effects of Regadenoson include chest pain and shortness of breath. Other common AEs include bradycardia, hypotension, and even asystole in some patients, potentially linked to vagal stimulation [19]. Clinicians using Regadenoson should evaluate the risk of vagal stimulation in each individual and proceed with caution in patients who may not tolerate prolonged hypotension, such as those with severe left ventricular dysfunction or cerebrovascular disease. Atropine should be considered for managing bradycardia or hypotension that does not respond to simple interventions, such as head-down positioning or gentle leg exercise [19]. Additionally, the drug could potentially lead to electrocardiogram changes such as ST-segment elevation [20] and QTc prolongation [21,22]. In more severe cases, complications such as myocardial infarction, multi-vessel coronary artery thrombosis [23,24], heart conduction block [25,26,27], and cardiac arrest [25,27,28,29] have been reported. Agrawal et al. further highlighted the potential for coronary steal syndrome with Regadenoson, which presented as chest pain during MPI testing, with symptoms worsening upon the administration of oral nitroglycerin [30]. In a similar case, chest pain improved with the use of theophylline. Thus, the cardiovascular side effects of Regadenoson reported in the FAERS database are consistent with observations from previous research. It is essential to accurately identify these side effects and implement appropriate treatment measures.

The nervous system disorders induced by Regadenoson primarily include headache, seizure, dizziness, loss of consciousness, tremor, unresponsive to stimuli, syncope, presyncope, aphasia, and transient ischemic attack. These AEs on the nervous system are consistent with the findings in our study [31,32,33]. Robert et al. reported three cases of seizures induced by Regadenoson, whereas there are currently no reports linking adenosine itself to seizures, likely because adenosine provides protective effects to the central nervous system. The reasons may be as follows. The limited expression and restricted distribution of central adenosine A_2A_ receptors suggested that the activation of the A_2A_ receptor by Regadenoson could potentially contribute to abnormal glutamatergic excitability, which might reduce the neuroprotective effects mediated by A_1_ receptors [34].

These mechanisms might explain the association between Regadenoson and seizures reported in some cases. Therefore, Regadenoson should be avoided in patients with uncontrolled epilepsy, recent seizures, or a history of epilepsy, even if their condition is well controlled. It is also important to note that theophylline, commonly used to counteract the side effects of vasodilators, may lower the seizure threshold and prolong the duration of seizures, indicating that it should also be avoided [35]. Clinically, benzodiazepines are frequently used to terminate seizures.

The side effects of Regadenoson not mentioned on the label include renal and urinary disorders such as micturition urgency, psychiatric disorders like mental status changes and conversion disorder, eye disorders such as eye movement disorder, as well as reproductive system and breast disorders like genital paraesthesia. These potential adverse effects, which are not listed on the drug label, may also impact the clinical use of Regadenoson. These side effects could potentially lead to physiological and psychological disturbances in some patients. Therefore, it is essential to monitor the reactions and conditions of patients after Regadenoson use. Effective treatment is also an important measure to improve these rare adverse effects.

This study also performed a temporal analysis of the AEs associated with Regadenoson. The results revealed that adverse reactions may occur shortly after drug administration, with most reactions occurring within a relatively short time. These findings highlight the importance of vigilant monitoring and the need to establish an effective timeline for tracking drug-related adverse reactions. Early monitoring is particularly critical for patients receiving Regadenoson, as it may help identify and manage potential side effects, potentially improving patient safety and treatment outcomes.

Clinicians should consider the following key points to mitigate the potential risks for the management of Regadenoson. First, baseline assessments including blood pressure, heart rate, and electrocardiogram (ECG), as well as patients with a history of cardiovascular disease, asthma, or seizures, should be carefully evaluated before administering Regadenoson. Those with severe left ventricular dysfunction, left ventricular outflow obstruction, or cerebrovascular disease require a thorough assessment prior to the use of Regadenoson. In addition, elderly patients may be more susceptible to the adverse effects of Regadenoson. Thus, patients’ hemodynamic stability and overall clinical conditions before administering Regadenoson should be assessed by physicians. Second, continuous monitoring of heart rate and blood pressure during and after Regadenoson administration is essential to detect early signs of adverse reactions. Ensuring that emergency medications, such as aminophylline (which can reverse the effects of Regadenoson) and atropine (for managing severe bradycardia or hypotension that does not respond to simple interventions), as well as other necessary equipment, are readily available and accessible during the administration of Regadenoson is important. Third, physicians should stay updated with the latest literature and guidelines on Regadenoson use, particularly in high-risk populations. Further studies are needed to identify predictive biomarkers or clinical features that could help stratify patients at higher risk for AEs.

In clinical practice, non-invasive imaging techniques enable the evaluation of coronary anatomy via coronary computed tomography angiography (CCTA) and the assessment of inducible myocardial ischemia through functional stress tests, such as stress echocardiography, cardiac magnetic resonance imaging (CMR), SPECT, or PET. In cases where vasodilators (including Regadenoson) are contraindicated, dobutamine stress cardiac magnetic resonance imaging (Dobutamine stress-CMR) serves as an effective alternative for assessing myocardial ischemia, particularly in patients with severe renal disease or contraindications to gadolinium-based contrast agents. In the future, emerging techniques such as blood oxygen level-dependent (BOLD) CMR and hybrid imaging (e.g., PET/CMR) hold promise for expanding the application of CMR in ischemic heart disease, combining functional and anatomical imaging advantages to enhance diagnostic accuracy [36].

This study has several limitations. First, the FAERS database is a spontaneous reporting system used by doctors, pharmacists, and drug users, which can lead to missing or inaccurate data. For example, some reporters may not have uploaded their reports to the system, leading to missing data. However, we have implemented the following measures to support the reliability of the experimental results. First, we implemented a rigorous data cleaning process to mitigate the impact of potential inaccuracies, including removing duplicate reports, correcting obvious data entry errors, and excluding incomplete or unreliable records. Second, the large sample size of our study helps to offset the potential impact of individual data inaccuracies. The substantial data enhance the statistical power, allowing us to draw meaningful conclusions. Third, given the limited data from this study, there is a need for the further expansion of the sample size and the continuous growth of the database to explore the clinical side effects of the drug. Fourth, since Regadenoson was first introduced in 2008, 98% of the data in the FAERS database come from the United States, with only a small proportion of data from countries like the United Kingdom, Germany, and Sweden, which may lead to reporting bias. Fifth, the FAERS database does not consistently provide detailed clinical information, such as the hemodynamic status of patients at the time of Regadenoson administration, which makes it challenging to draw definitive conclusions about its use in unstable conditions. Sixth, FAERS is more suitable for identifying potential risk signals rather than providing precise epidemiological data.

Overall, Regadenoson serves as a crucial agent in non-invasive cardiac imaging, providing valuable information for the diagnosis and management of coronary artery disease and other heart conditions.

## 5. Conclusions

This study performed a comprehensive and systematic analysis of AEs associated with Regadenoson by utilizing the FAERS database, focusing on all relevant reports since its approval in 2008. The analysis supported the presence of known AEs and suggested the possibility of additional AEs not currently listed on the product label, such as micturition urgency, mental status changes, conversion disorder, eye movement disorder, and genital paraesthesia. These findings provide essential safety considerations for the clinical use of Regadenoson and underscore the necessity of careful patient monitoring. Given the potential risks, the safety profile of Regadenoson should be subject to ongoing surveillance to ensure its safe application in clinical practice.

## Figures and Tables

**Table 1:** Clinical characteristics of AE reports related to Regadenoson from the FAERS database (Q1 2004–Q3 2024).

Characteristics	Number of Cases	Proportion of Cases (%)
Number of AE reports	4408	
Number of AEs induced by Regadenoson	10,663	
Sex		
Male	1430	47.75
Female	2105	32.44
Not specified	873	19.8
Age		
Median (interquartile range)	69 (60, 78)	
<18	3	0.07
18–44	102	2.31
45–64	795	18.04
≥65	1573	35.69
Not specified	1935	43.9
Reporter		
Consumer	2279	51.7
Other health professionals	73	1.66
Pharmacist	1028	23.32
Physician	833	18.9
Not specified	195	4.42
Severity		
Severe		
Non-severe		
Reporting year		
2008	144	3.27
2009	101	2.29
2010	77	1.75
2011	45	1.02
2012	394	8.94
2013	534	12.11
2014	455	10.32
2015	287	6.51
2016	376	8.53
2017	387	8.78
1018	402	9.12
2019	304	6.9
2020	243	5.51
2021	155	3.52
2022	59	1.34
2023	95	2.16
2024	350	7.94
Top 5 reporting countries		
United States of America	4320	98
United Kingdom	28	0.64
Germany	8	0.18
Sweden	5	0.11
France	3	0.07

**Table 2:** Signal strength of Regadenoson-related AEs at the system organ class (SOC) level in the FAERS database.

System Organ Class (SOC)	Case Reports	ROR (95% CI)	PRR (95% CI)	PRR (χ^2^)	IC (IC025)	EBGM (EBGM05)
Nervous system disorders	2110	2.65 (2.53, 2.78)	2.33 (2.24, 2.42)	1744.07	1.22 (1.15)	2.33 (2.22)
General disorders and administration site conditions	1708	0.90 (0.86, 0.95)	0.92 (0.88, 0.96)	14.94	−0.12 (−0.20)	0.92 (0.87)
Gastrointestinal disorders	1359	1.57 (1.48, 1.66)	1.50 (1.43, 1.57)	246.15	0.58 (0.50)	1.50 (1.42)
Cardiac disorders	1336	5.28 (4.99, 5.60)	4.75 (4.52, 4.99)	4056.08	2.25 (2.16)	4.74 (4.48)
Investigations	826	1.28 (1.19, 1.38)	1.26 (1.18, 1.35)	47.15	0.33 (0.23)	1.26 (1.17)
Respiratory, thoracic and mediastinal disorders	693	1.41 (1.30, 1.52)	1.38 (1.29, 1.48)	76.60	0.47 (0.35)	1.38 (1.28)
Vascular disorders	534	2.42 (2.21, 2.64)	2.34 (2.16, 2.55)	420.65	1.23 (1.10)	2.34 (2.15)
Musculoskeletal and connective tissue disorders	526	0.95 (0.87, 1.04)	0.95 (0.88, 1.04)	1.31	−0.07 (−0.20)	0.95 (0.87)
Injury, poisoning, and procedural complications	452	0.38 (0.35, 0.42)	0.41 (0.37, 0.45)	427.33	−1.29 (−1.42)	0.41 (0.37)
Skin and subcutaneous tissue disorders	309	0.52 (0.47, 0.59)	0.54 (0.48, 0.60)	128.95	−0.89 (−1.06)	0.54 (0.48)
Psychiatric disorders	211	0.34 (0.29, 0.39)	0.35 (0.31, 0.40)	269.95	−1.51 (−1.71)	0.35 (0.31)
Product issues	157	0.91 (0.78, 1.07)	0.91 (0.78, 1.07)	1.32	−0.13 (−0.36)	0.91 (0.78)
Eye disorders	129	0.60 (0.51, 0.72)	0.61 (0.51, 0.72)	33.31	−0.72 (−0.97)	0.61 (0.51)
Immune system disorders	113	0.96 (0.80, 1.16)	0.96 (0.80, 1.16)	0.15	−0.05 (−0.32)	0.96 (0.80)
Renal and urinary disorders	52	0.25 (0.19, 0.33)	0.26 (0.19, 0.34)	115.00	−1.97 (−2.35)	0.26 (0.19)
Infections and infestations	30	0.05 (0.04, 0.07)	0.05 (0.04, 0.08)	527.41	−4.22 (−4.69)	0.05 (0.04)
Metabolism and nutrition disorders	24	0.10 (0.07, 0.15)	0.10 (0.07, 0.15)	190.11	−3.27 (−3.79)	0.10 (0.07)
Surgical and medical procedures	21	0.14 (0.09, 0.22)	0.15 (0.10, 0.22)	105.75	−2.77 (−3.33)	0.15 (0.10)
Social circumstances	18	0.36 (0.23, 0.58)	0.36 (0.23, 0.58)	20.18	−1.46 (−2.07)	0.36 (0.23)
Reproductive system and breast disorders	15	0.16 (0.09, 0.26)	0.16 (0.10, 0.26)	68.16	−2.67 (−3.31)	0.16 (0.09)
Ear and labyrinth disorders	14	0.30 (0.18, 0.51)	0.30 (0.18, 0.51)	22.67	−1.73 (−2.40)	0.30 (0.18)
Blood and lymphatic system disorders	9	0.05 (0.03, 0.09)	0.05 (0.03, 0.10)	165.00	−4.32 (−5.09)	0.05 (0.03)
Hepatobiliary disorders	6	0.06 (0.03, 0.14)	0.06 (0.03, 0.14)	86.99	−4.03 (−4.91)	0.06 (0.03)
Congenital, familial, and genetic disorders	4	0.12 (0.05, 0.33)	0.12 (0.05, 0.33)	24.80	−3.01 (−4.02)	0.12 (0.05)
Neoplasms benign, malignant, and unspecified (incl cysts and polyps)	4	0.01 (0.01, 0.04)	0.01 (0.01, 0.04)	280.62	−6.13 (−7.11)	0.01 (0.01)
Endocrine disorders	3	0.11 (0.04, 0.34)	0.11 (0.04, 0.34)	21.44	−3.17 (−4.25)	0.11 (0.04)

Note: ranked by case reports.

**Table 3:** Top 50 most frequent AEs for Regadenoson at the preferred term (PT) level.

Preferred Term (PT)	Case Reports	ROR (95% CI)	PRR (95% CI)	PRR (χ^2^)	IC (IC025)	EBGM (EBGM05)
Nausea	483	3.68	3.55	897.56	1.83	3.55
Dyspnea	375	3.93	3.83	789.54	1.94	3.82
Injection site extravasation	355	162.23	156.87	53,349.2	7.25	152.21
Vomiting	344	4.41	4.3	876.18	2.1	4.29
Headache	291	2.72	2.67	307.35	1.42	2.67
Hypotension	289	8.52	8.31	1862.23	3.05	8.3
Cardiac arrest	284	19.93	19.43	4951.44	4.27	19.36
Seizure	282	9.59	9.36	2109.03	3.22	9.35
Dizziness	246	2.89	2.85	296.6	1.51	2.84
Chest pain	205	6.35	6.25	905.49	2.64	6.24
Blood pressure decreased	201	17.75	17.43	3106.12	4.12	17.38
Bradycardia	192	20.69	20.33	3518.35	4.34	20.26
Loss of consciousness	176	7.97	7.86	1053.63	2.97	7.85
Tremor	166	5.74	5.66	638.21	2.5	5.66
Unresponsive to stimuli	149	33.79	33.34	4645.13	5.05	33.13
Incorrect product administration duration	131	14.97	14.8	1681.96	3.88	14.76
Pain in extremity	131	2.53	2.51	119.33	1.33	2.51
Heart rate increased	116	6.82	6.76	568.99	2.75	6.75
Heart rate decreased	100	16.19	16.05	1407.55	4	16
Syncope	99	5.62	5.58	372.16	2.48	5.57
Chest discomfort	96	5.57	5.53	356.72	2.47	5.53
Hyperhidrosis	82	3.62	3.6	154.35	1.85	3.6
Blood pressure increased	78	2.93	2.92	98.6	1.54	2.92
Atrioventricular block	75	56.4	56.01	4008.67	5.79	55.41
Atrioventricular block complete	74	64.05	63.61	4504.58	5.97	62.84
Presyncope	66	16.07	15.97	923.9	3.99	15.93
Flushing	63	3.46	3.45	109.44	1.78	3.44
Infusion site extravasation	58	54.33	54.04	2988.11	5.74	53.49
Atrial fibrillation	50	2.95	2.94	64.02	1.55	2.94
Extravasation	46	63.85	63.58	2798.75	5.97	62.81
Atrioventricular block second degree	44	82.72	82.39	3481.41	6.34	81.09
Wheezing	42	4.43	4.42	111.17	2.14	4.42
Sinus arrest	40	153.34	152.77	5855.58	7.21	148.35
Electrocardiogram ST segment depression	36	91.88	91.57	3168.08	6.49	89.97
Acute myocardial infarction	36	6.72	6.7	174.37	2.74	6.69
Cardio-respiratory arrest	36	4.75	4.74	106.18	2.24	4.74
Respiratory arrest	35	6.88	6.86	175.19	2.78	6.86
Retching	32	8.79	8.76	219.78	3.13	8.75
Pallor	31	6.42	6.41	141.36	2.68	6.4
Aphasia	31	5.73	5.72	120.59	2.51	5.71
Product administration error	31	3.39	3.38	51.95	1.76	3.38
Bronchospasm	28	11.1	11.08	256.22	3.47	11.06
Ventricular extrasystoles	27	14.45	14.42	336.34	3.85	14.38
Electrocardiogram ST segment elevation	26	43.84	43.73	1076.46	5.44	43.37
Electrocardiogram abnormal	25	17.67	17.63	390.97	4.14	17.58
Throat tightness	25	5.36	5.35	88.31	2.42	5.34
Infusion site pain	24	11.8	11.77	236.09	3.55	11.75
Nodal rhythm	23	87.83	87.64	1936.83	6.43	86.18
Ventricular tachycardia	23	7.95	7.94	139.33	2.99	7.93
Supraventricular tachycardia	22	13.02	12.99	242.98	3.7	12.96

**Table 4:** Representative classic and non-classic AEs of Regadenoson and their clinical implications.

Adverse Effect Type	Classic Adverse Effects	Non-Classic Adverse Effects	Clinical Implications
Cardiovascular	- Chest pain - ST-segment depression	- Severe hypotension - Atrioventricular block	- Monitor ECG and blood pressure closely - Have emergency medications (e.g., aminophylline) ready
Respiratory	- Dyspnea - Bronchospasm	- Respiratory arrest	- Use with caution in asthma/COPD patients - Ensure immediate access to bronchodilators
Neurological	- Headache - Dizziness	- Seizures	- Assess patient history for seizure disorders - Monitor for neurological symptoms
Gastrointestinal	- Abdominal discomfort - Nausea	- Severe vomiting	- Provide symptomatic treatment - Ensure hydration
Systemic	- Flushing - Feeling of warmth	- Anaphylaxis	- Monitor for signs of hypersensitivity - Have emergency protocols in place.
Other	- Metallic taste	- Acute kidney injury	- Assess renal function before administration - Monitor hydration status

## Data Availability

The original contributions presented in the study are included in the article; further inquiries can be directed to the corresponding author.

## Supplementary Materials

The following supporting information can be downloaded at: https://www.mdpi.com/article/10.3390/jcm14061860/s1.

## Abbreviations

The following abbreviations are used in this manuscript:

MPI	myocardial perfusion imaging
AE	adverse event
FAERS	FDA Adverse Event Reporting System
ROR	reporting odds ratio
PRR	proportional reporting ratio
MGPS	multi-item gamma Poisson shrinker
BCPNN	Bayesian confidence propagation neural network
MBF	myocardial blood flow
CAD	coronary artery disease
FDA	Food and Drug Administration
CASEIDs	case identifiers
MedDRA	Medical Dictionary for Regulatory Activities
PT	preferred term
SOC	system organ class
MHRA	Medicines and Healthcare products Regulatory Agency
IQR	interquartile range
DEMO	demographic data
REAC	reaction data
PET	positron emission tomography
SPECT	single-photon emission computed tomography (SPECT) and (PET
ECG	electrocardiogram
CCTA	coronary computed tomography angiography
CMR	cardiac magnetic resonance imaging
BOLD	blood oxygen level-dependent
