# Supplementary material for: Assessing the Real-World Safety of Regadenoson for Myocardial Perfusion Imaging: Insights from a Comprehensive Analysis of FAERS Data
Source: J Clin Med. 2025 Mar 10;14(6):1860. doi: 10.3390/jcm14061860 (PMC11943247; doi:10.3390/jcm14061860)
Supplement: Supplementary file 1 [file jcm-14-01860-s001.zip › jcm-3473641-supplementary.pdf]

Supplementary Material

Supplementary Tables

Supplementary Table S1: Two-by-two contingency table for disproportionality analyses

	Target AEs	Other AEs	Total
Regadenoson	a	b	a+b
Other drugs	c	d	c+d
Total	a+c	c+d	a+b+c+d

Abbreviations: AEs, adverse events; a, the number of reports containing target AEs caused by Regadenoson; b, the number of reports containing other AEs caused by Regadenoson; c, the number of reports containing target AEs caused by other drugs; b, the number of reports containing other AEs caused by other drugs.

Supplementary Table S2: Five major algorithms used for signal detection.

Regadenoson	Equation	Criteria
ROR	$ROR = \frac{(a/c)}{(b/d)} = \frac{ad}{bc}$<br>95%CI=$e^{\ln(ROR)\pm1.96(1/a+1/b+1/c+1/d)^{\wedge}0.5}$	N≥3 and the 95% CI lower limit>1
PRR	$PRR = \frac{a/(a+b)}{c/(c+d)}$<br>$\chi 2 = \frac{(ad - bc)^2(a+b+c+d)}{(a+b)(a+c)(c+d)(b+d)}$	PRR (95% CI): N≥3 and the 95% CI lower limit>1<br>PRR (χ^2): N≥3, PRR≥2 and χ^2≥4
BCPNN	IC=$log_2 \frac{p(x,y)}{p(x)p(y)} = log_2 \frac{a(a+b+c+d)}{(a+b)(a+c)}$<br>E(IC)=$log_2 \frac{(a+\gamma 11)(a+b+c+d+\alpha)(a+b+c+d+\beta)}{(a+b+c+d+\gamma)(a+b+\alpha 1)(a+c+\beta 1)}$<br>V(IC)=$\frac{1}{(ln2)^2}\{[\frac{(a+b+c+d)-a+\gamma-\gamma 11}{(a+\gamma 11)(1+a+b+c+d+\gamma)}] + [\frac{(a+b+c+d)-(a+b)+\alpha-\alpha 1}{(a+b+\alpha 1)(1+a+b+c+d+\alpha)}] + [\frac{(a+b+c+d)-(a+c)+\beta-\beta 1}{(a+c+\beta 1)(1+a+b+c+d+\beta)}]\}$<br>$\gamma = \gamma 11 \frac{(a+b+c+d+\alpha)(a+b+c+d+\beta)}{(a+b+\alpha 1)(a+c+\beta 1)}$<br>IC-2SD=E(IC)-2$\sqrt{V(IC)}$	Lower limit of IC025>0

	$\alpha1=\beta1$, $\alpha=\beta=2$	
MGPS	$\text{EBGM} = \frac{a(a+b+c+d)}{(a+c)(a+b)}$<br>$95\%\text{CI} = e^{\ln(\text{EBGM})\pm 1.96\sqrt{(\frac{1}{a}+\frac{1}{b}+\frac{1}{c}+\frac{1}{d})}}$	EBGM05>2

Abbreviations: AEs, adverse events; a, the number of reports containing target AEs caused by Regadenoson; b, the number of reports containing other AEs caused by Regadenoson; c, the number of reports containing target AEs caused by other drugs; b, the number of reports containing other AEs caused by other drugs; CI, confidence interval; N, the number of reports; χ^2, chi-squared; IC, information component; IC025, the lower limit of 95% CI of the IC; E(IC), the IC expectations; V(IC), the variance of IC; EBGM, empirical Bayesian geometric mean; EBGM05, the lower limit of 95% CI of EBGM.

Supplementary Table S3 Signal strength of AEs at the Preferred Term (PT) level ranked by FAERS data.

Preferred Term (PT)	Case reports	ROR (95% CI)	PRR (95% CI)	PRR (χ^2)	IC (IC025)	EBGM (EBGM05)
Nausea	483	3.68(3.36,4.03)	3.55(3.26,3.88)	897.56	1.83(1.69)	3.55(3.24)
Dyspnoea	375	3.93(3.54,4.36)	3.83(3.46,4.23)	789.54	1.94(1.77)	3.82(3.45)
Injection site extravasation	355	162.23(145.72,180.62)	156.87(141.39,174.04)	53349.2	7.25(6.58)	152.21(136.72)
Vomiting	344	4.41(3.96,4.91)	4.30(3.87,4.77)	876.18	2.10(1.93)	4.29(3.86)
Headache	291	2.72(2.42,3.05)	2.67(2.39,2.99)	307.35	1.42(1.24)	2.67(2.38)
Hypotension	289	8.52(7.58,9.57)	8.31(7.42,9.31)	1862.23	3.05(2.85)	8.30(7.38)
Cardiac arrest	284	19.93(17.71,22.43)	19.43(17.32,21.79)	4951.44	4.27(4.01)	19.36(17.20)
Seizure	282	9.59(8.52,10.80)	9.36(8.35,10.51)	2109.03	3.22(3.01)	9.35(8.31)
Dizziness	246	2.89(2.55,3.28)	2.85(2.51,3.22)	296.6	1.51(1.31)	2.84(2.51)
Chest pain	205	6.35(5.53,7.29)	6.25(5.46,7.16)	905.49	2.64(2.40)	6.24(5.44)

Blood pressure decreased	201	17.75(15.43,20.41)	17.43(15.20,20.00)	3106.12	4.12(3.80)	17.38(15.11)
Bradycardia	192	20.69(17.93,23.87)	20.33(17.67,23.40)	3518.35	4.34(3.99)	20.26(17.56)
Loss of consciousness	176	7.97(6.87,9.25)	7.86(6.78,9.10)	1053.63	2.97(2.70)	7.85(6.76)
Tremor	166	5.74(4.92,6.69)	5.66(4.87,6.58)	638.21	2.50(2.23)	5.66(4.85)
Unresponsive to stimuli	149	33.79(28.73,39.75)	33.34(28.41,39.12)	4645.13	5.05(4.53)	33.13(28.17)
Incorrect product administration duration	131	14.97(12.60,17.79)	14.80(12.48,17.55)	1681.96	3.88(3.49)	14.76(12.42)
Pain in extremity	131	2.53(2.13,3.00)	2.51(2.12,2.97)	119.33	1.33(1.06)	2.51(2.11)
Heart rate increased	116	6.82(5.68,8.19)	6.76(5.64,8.10)	568.99	2.75(2.42)	6.75(5.62)
Heart rate decreased	100	16.19(13.29,19.72)	16.05(13.20,19.51)	1407.55	4.00(3.51)	16.00(13.14)
Syncope	99	5.62(4.61,6.85)	5.58(4.58,6.79)	372.16	2.48(2.12)	5.57(4.57)
Chest discomfort	96	5.57(4.56,6.82)	5.53(4.53,6.75)	356.72	2.47(2.11)	5.53(4.52)
Hyperhidrosis	82	3.62(2.91,4.50)	3.60(2.90,4.47)	154.35	1.85(1.49)	3.60(2.90)

Blood pressure increased	78	2.93(2.35,3.67)	2.92(2.34,3.64)	98.6	1.54(1.18)	2.92(2.34)
Atrioventricular block	75	56.40(44.89,70.87)	56.01(44.65,70.27)	4008.67	5.79(4.68)	55.41(44.10)
Atrioventricular block complete	74	64.05(50.89,80.61)	63.61(50.62,79.94)	4504.58	5.97(4.77)	62.84(49.92)
Presyncope	66	16.07(12.61,20.47)	15.97(12.55,20.33)	923.9	3.99(3.35)	15.93(12.50)
Flushing	63	3.46(2.70,4.43)	3.45(2.69,4.41)	109.44	1.78(1.37)	3.44(2.69)
Infusion site extravasation	58	54.33(41.92,70.42)	54.04(41.75,69.95)	2988.11	5.74(4.44)	53.49(41.26)
Atrial fibrillation	50	2.95(2.23,3.89)	2.94(2.23,3.88)	64.02	1.55(1.10)	2.94(2.22)
Extravasation	46	63.85(47.71,85.45)	63.58(47.57,84.99)	2798.75	5.97(4.34)	62.81(46.93)
Atrioventricular block second degree	44	82.72(61.38,111.49)	82.39(61.20,110.90)	3481.41	6.34(4.43)	81.09(60.17)
Wheezing	42	4.43(3.27,6.00)	4.42(3.27,5.98)	111.17	2.14(1.59)	4.42(3.26)
Sinus arrest	40	153.34(111.90,210.13)	152.77(111.61,209.11)	5855.58	7.21(4.55)	148.35(108.25)
Electrocardiogram ST segment depression	36	91.88(66.04,127.81)	91.57(65.89,127.25)	3168.08	6.49(4.24)	89.97(64.67)

Acute myocardial infarction	36	6.72(4.84,9.32)	6.70(4.83,9.28)	174.37	2.74(2.06)	6.69(4.82)
Cardio-respiratory arrest	36	4.75(3.43,6.59)	4.74(3.42,6.57)	106.18	2.24(1.63)	4.74(3.41)
Respiratory arrest	35	6.88(4.94,9.59)	6.86(4.93,9.56)	175.19	2.78(2.08)	6.86(4.92)
Retching	32	8.79(6.21,12.44)	8.76(6.20,12.39)	219.78	3.13(2.32)	8.75(6.18)
Pallor	31	6.42(4.51,9.14)	6.41(4.51,9.11)	141.36	2.68(1.94)	6.40(4.50)
Aphasia	31	5.73(4.03,8.16)	5.72(4.02,8.13)	120.59	2.51(1.80)	5.71(4.01)
Product administration error	31	3.39(2.38,4.82)	3.38(2.38,4.80)	51.95	1.76(1.14)	3.38(2.37)
Bronchospasm	28	11.10(7.66,16.10)	11.08(7.65,16.04)	256.22	3.47(2.50)	11.06(7.63)
Ventricular extrasystoles	27	14.45(9.90,21.10)	14.42(9.89,21.03)	336.34	3.85(2.74)	14.38(9.85)
Electrocardiogram ST segment elevation	26	43.84(29.78,64.52)	43.73(29.74,64.31)	1076.46	5.44(3.52)	43.37(29.47)
Electrocardiogram abnormal	25	17.67(11.93,26.18)	17.63(11.91,26.10)	390.97	4.14(2.86)	17.58(11.86)
Throat tightness	25	5.36(3.62,7.93)	5.35(3.61,7.91)	88.31	2.42(1.63)	5.34(3.61)

Infusion site pain	24	11.80(7.90,17.62)	11.77(7.89,17.57)	236.09	3.55(2.46)	11.75(7.87)
Nodal rhythm	23	87.83(58.14,132.70)	87.64(58.06,132.30)	1936.83	6.43(3.65)	86.18(57.04)
Ventricular tachycardia	23	7.95(5.28,11.98)	7.94(5.28,11.95)	139.33	2.99(2.03)	7.93(5.26)
Supraventricular tachycardia	22	13.02(8.56,19.79)	12.99(8.55,19.74)	242.98	3.70(2.49)	12.96(8.53)
Seizure like phenomena	21	78.82(51.20,121.34)	78.67(51.15,121.01)	1585.89	6.28(3.49)	77.49(50.34)
Pulse absent	21	24.40(15.89,37.48)	24.36(15.87,37.38)	468.12	4.60(2.94)	24.24(15.78)
Syringe issue	20	6.91(4.46,10.72)	6.90(4.45,10.70)	100.82	2.79(1.80)	6.89(4.44)
Pain in jaw	20	3.96(2.56,6.15)	3.96(2.55,6.13)	44.22	1.98(1.16)	3.96(2.55)
Transient ischaemic attack	20	3.29(2.12,5.10)	3.28(2.12,5.09)	31.73	1.71(0.93)	3.28(2.12)
Sinus bradycardia	19	11.12(7.09,17.45)	11.10(7.08,17.41)	174.35	3.47(2.23)	11.08(7.06)
Anal incontinence	19	8.09(5.16,12.70)	8.08(5.15,12.67)	117.7	3.01(1.93)	8.07(5.14)
Mental status changes	19	3.89(2.48,6.11)	3.89(2.48,6.09)	40.73	1.96(1.12)	3.88(2.48)
Ventricular fibrillation	17	8.67(5.38,13.96)	8.66(5.38,13.93)	114.96	3.11(1.92)	8.64(5.37)

Generalised tonic-clonic seizure	17	3.76(2.34,6.06)	3.76(2.34,6.05)	34.44	1.91(1.02)	3.76(2.34)
Feeling cold	17	3.48(2.16,5.60)	3.47(2.16,5.59)	29.95	1.80(0.93)	3.47(2.16)
Urinary incontinence	17	3.26(2.03,5.25)	3.26(2.03,5.24)	26.62	1.70(0.85)	3.26(2.02)
Cold sweat	16	5.17(3.16,8.44)	5.16(3.16,8.43)	53.66	2.37(1.35)	5.16(3.16)
Atrioventricular block first degree	15	18.82(11.33,31.26)	18.80(11.32,31.20)	251.82	4.23(2.43)	18.73(11.28)
Arteriospasm coronary	14	21.26(12.57,35.94)	21.23(12.56,35.87)	268.76	4.40(2.43)	21.14(12.50)
Bundle branch block left	14	18.07(10.69,30.54)	18.04(10.68,30.48)	224.61	4.17(2.33)	17.98(10.64)
Blood pressure systolic decreased	14	17.95(10.62,30.34)	17.93(10.61,30.29)	222.98	4.16(2.32)	17.87(10.57)
Product packaging quantity issue	14	8.82(5.22,14.91)	8.81(5.22,14.88)	96.79	3.14(1.79)	8.80(5.21)
Myocardial ischaemia	14	6.55(3.88,11.07)	6.54(3.87,11.05)	65.67	2.71(1.51)	6.54(3.87)
Hemiparesis	13	4.26(2.47,7.34)	4.26(2.47,7.33)	32.39	2.09(1.02)	4.26(2.47)
Electrocardiogram change	11	64.71(35.69,117.31)	64.64(35.67,117.12)	680.59	6.00(2.52)	63.84(35.21)
Eye movement disorder	11	8.21(4.55,14.84)	8.21(4.54,14.82)	69.51	3.03(1.52)	8.20(4.53)

Facial paralysis	11	4.39(2.43,7.93)	4.38(2.43,7.92)	28.72	2.13(0.94)	4.38(2.43)
Contrast media reaction	10	24.61(13.22,45.82)	24.59(13.21,45.76)	225.22	4.61(2.09)	24.48(13.15)
Supraventricular extrasystoles	10	16.25(8.73,30.23)	16.23(8.73,30.19)	142.48	4.02(1.89)	16.18(8.70)
Pulseless electrical activity	10	11.71(6.30,21.79)	11.70(6.29,21.76)	97.68	3.55(1.70)	11.68(6.28)
Electrocardiogram ST segment abnormal	9	63.47(32.88,122.52)	63.42(32.87,122.35)	546.14	5.97(2.21)	62.65(32.46)
Vein rupture	9	32.92(17.09,63.42)	32.89(17.08,63.34)	276.54	5.03(2.05)	32.69(16.97)
Administration site extravasation	8	52.09(25.95,104.56)	52.05(25.95,104.43)	396.54	5.69(1.99)	51.54(25.68)
Vomiting projectile	8	17.66(8.82,35.37)	17.65(8.82,35.33)	125.24	4.14(1.66)	17.59(8.79)
Muscle contractions involuntary	8	12.73(6.36,25.49)	12.73(6.36,25.46)	86.22	3.67(1.50)	12.70(6.34)
Ischaemia	8	9.54(4.76,19.09)	9.53(4.76,19.06)	60.97	3.25(1.33)	9.51(4.75)
Procedural complication	8	5.69(2.84,11.38)	5.68(2.84,11.37)	30.85	2.51(0.94)	5.68(2.84)
Injection site discomfort	8	4.90(2.45,9.79)	4.89(2.45,9.78)	24.75	2.29(0.81)	4.89(2.44)

Micturition urgency	8	4.15(2.07,8.30)	4.15(2.07,8.29)	19.09	2.05(0.66)	4.14(2.07)
Psychogenic seizure	7	44.95(21.35,94.61)	44.92(21.35,94.50)	297.95	5.48(1.76)	44.53(21.16)
Device connection issue	7	26.40(12.56,55.49)	26.38(12.55,55.43)	170.04	4.71(1.64)	26.25(12.49)
Kounis syndrome	7	19.09(9.09,40.12)	19.08(9.09,40.07)	119.5	4.25(1.52)	19.01(9.05)
Sinus node dysfunction	7	13.04(6.21,27.38)	13.03(6.21,27.35)	77.55	3.70(1.36)	13.00(6.19)
Bundle branch block right	7	8.37(3.99,17.57)	8.36(3.98,17.55)	45.3	3.06(1.10)	8.35(3.98)
Infusion site swelling	7	7.51(3.58,15.77)	7.51(3.58,15.75)	39.42	2.91(1.03)	7.50(3.57)
Stress cardiomyopathy	7	7.46(3.55,15.66)	7.46(3.55,15.65)	39.08	2.90(1.02)	7.45(3.55)
Acute pulmonary oedema	7	7.04(3.35,14.77)	7.03(3.35,14.76)	36.18	2.81(0.98)	7.02(3.35)
Incoherent	7	6.00(2.86,12.60)	6.00(2.86,12.58)	29.12	2.58(0.86)	5.99(2.85)
Hemiplegic migraine	6	125.35(55.75,281.83)	125.28(55.75,281.55)	721.99	6.93(1.63)	122.30(54.40)
Physical product label issue	6	33.28(14.91,74.29)	33.26(14.91,74.22)	186.55	5.05(1.47)	33.05(14.81)
Blood pressure immeasurable	6	21.78(9.77,48.58)	21.77(9.77,48.54)	118.41	4.44(1.36)	21.68(9.72)

Heart rate abnormal	6	6.48(2.91,14.43)	6.48(2.91,14.42)	27.74	2.69(0.77)	6.47(2.90)
False negative investigation result	5	41.92(17.38,101.09)	41.90(17.38,101.01)	197.98	5.38(1.24)	41.56(17.23)
Immediate post-injection reaction	5	25.13(10.44,60.53)	25.12(10.44,60.48)	115.25	4.64(1.14)	25.00(10.38)
Inappropriate affect	5	17.14(7.12,41.25)	17.13(7.12,41.21)	75.7	4.09(1.03)	17.08(7.10)
Stridor	5	9.74(4.05,23.43)	9.74(4.05,23.41)	39.13	3.28(0.81)	9.72(4.04)
Hyperventilation	5	4.84(2.01,11.63)	4.83(2.01,11.62)	15.19	2.27(0.38)	4.83(2.01)
Cardiac perfusion defect	4	536.50(191.45,1503.47)	536.30(191.44,1502.38)	1933.66	8.92(0.95)	485.32(173.18)
Injection related reaction	4	16.05(6.01,42.84)	16.05(6.01,42.81)	56.26	4.00(0.71)	16.00(5.99)
Suspected product quality issue	4	15.80(5.92,42.18)	15.80(5.92,42.15)	55.27	3.98(0.70)	15.75(5.90)
Conversion disorder	4	11.45(4.29,30.56)	11.45(4.29,30.53)	38.06	3.51(0.60)	11.43(4.28)
Electrocardiogram T wave inversion	4	9.83(3.68,26.22)	9.83(3.69,26.20)	31.65	3.29(0.54)	9.81(3.68)
Coronary steal syndrome	3	1274.07(359.48,4515.57)	1273.71(359.48,4513.01)	3052.1	9.99(0.38)	1019.17(287.56)
Genital paraesthesia	3	61.65(19.74,192.49)	61.63(19.74,192.37)	176.8	5.93(0.48)	60.91(19.51)

Left ventricle outflow tract obstruction	3	49.80(15.97,155.29)	49.79(15.97,155.20)	142.03	5.62(0.46)	49.31(15.81)
Cardiac procedure complication	3	26.45(8.50,82.27)	26.44(8.50,82.22)	73.07	4.72(0.40)	26.31(8.46)
Moaning	3	15.71(5.06,48.81)	15.71(5.06,48.78)	41.19	3.97(0.30)	15.66(5.04)
Sinus arrhythmia	3	12.55(4.04,38.98)	12.55(4.04,38.96)	31.81	3.65(0.24)	12.52(4.03)
Contrast media allergy	3	11.88(3.83,36.89)	11.88(3.83,36.87)	29.81	3.57(0.23)	11.85(3.82)
Conduction disorder	3	10.02(3.23,31.10)	10.02(3.23,31.09)	24.3	3.32(0.18)	10.00(3.22)
Tunnel vision	3	9.38(3.02,29.12)	9.38(3.02,29.10)	22.41	3.23(0.15)	9.36(3.02)
Slow speech	3	7.45(2.40,23.11)	7.44(2.40,23.10)	16.71	2.89(0.07)	7.44(2.40)

Abbreviation: PT, preferred term; ROR, reporting odds ratio; CI, confidence interval; PRR, proportional reporting ratio; χ^2, Chi-Square; IC, information component; IC025, the lower limit of the 95% CI of the IC; EBGM, empirical Bayesian geometric mean; EBGM05, the lower limit of the 95% CI of EBGM. Signals are detected when all the following criteria are met: N≥3, PRR≥2 and Chi-Square (χ^2) ≥4, lower limit of 95% CI of ROR>1, IC025>0, EBGM05>2.
